# Is the concept of personality capacious enough to incorporate virtues?

**DOI:** 10.3389/fpsyg.2023.1232637

**Published:** 2023-08-29

**Authors:** Blaine J. Fowers, Lukas F. Novak, Nona C. Kiknadze, Marah Selim

**Affiliations:** Department of Educational and Psychological Studies, University of Miami, Coral Gables, FL, United States

**Keywords:** personality, virtue, practical wisdom, agency, good life, individual differences, trait

## Abstract

We will consider four answers to the question about whether the concept of personality is capacious enough to incorporate virtues. The simplest is that the concept of personality encompasses all individual variations in persons. It follows from this answer that virtues would, as individual differences, be incorporated into personality. Unfortunately, definitions of personality do not always invoke such capaciousness, and, in practice, most scholars limit their work to the Big Five or HEXACO models, which do not incorporate virtues. The second answer is that the concept of personality incorporates all trait or dimension level variations across persons, with some exceptions, such as intelligence, attachment style, and psychopathy. Following this definition, virtues, as traits, would be incorporated into such a broad definition of personality. Unfortunately, the boundaries for inclusion and exclusion into personality are fuzzy in this case, and there is no extant definition of personality that solves this problem. The third answer is that personality traits and virtue traits are similar, but distinct concepts. This article presents conceptual and empirical arguments for this similarity in seeing traits as a higher order concept that includes the species of personality and the species of virtue. The fourth answer is that personality and virtue are unrelated. This answer is dismissed because there are many studies that indicate that they are correlated, and few advocate such a clear differentiation. The conclusion is that, pending conceptual and empirical results indicating otherwise, the genus-species relationship seems most fitting where traits are a genus, and personality and virtue are each a species within that genus.

## 1. Introduction

As the study of virtues has rapidly grown, the natural question of how virtue traits are related to personality has emerged clearly (Fowers et al., 2021). Given that both are frequently conceptualized as traits that have a significant degree of stability within persons (Roberts and Damian, 2019), differences between persons (John and Robins, 2022), responsiveness to circumstances (Lang et al., 2017), and are amenable to similar research methods (Jayawickreme and Fleeson, 2017; Fowers et al., 2021), it is reasonable to inquire about the degree of overlap or the possibility of subsuming one form of trait into the other. Because personality psychology is the more established research domain, the question is typically whether virtue traits are part of personality. The way that scholars decide to construe the relationship between personality and virtue has the potential to strongly shape one or both fields of study, so there is some urgency in clarifying the relationship.

We will consider four answers to the question about whether the concept of personality is capacious enough to incorporate virtues. The simplest answer is that the concept of personality encompasses all individual variations in persons, including virtues. This answer typically takes the form that virtues would, as individual differences, be incorporated into personality. Unfortunately, definitions of personality do not always invoke such capaciousness, and, in practice, most scholars limit their work to the Big Five or HEXACO models, which do not explicitly incorporate virtues. If virtues are to be incorporated into personality, there are four features of the virtue concept that must either be discarded or made to fit into personality theory. The inherent features of virtues are morality, agency, the aim of a good life (i.e., teleology), and practical wisdom. We discuss these features more fully below.

The second answer is that the concept of personality incorporates all trait or dimension level variations across persons, with some exceptions, such as intelligence, attachment style, and psychopathy. Following this definition, virtues, as traits, would be incorporated into such a broad definition of personality. One problem is that the exceptions render the boundaries for inclusion and exclusion into personality fuzzy, and there is no extant definition of personality that clarifies inclusion and exclusion. The misfit between widely accepted elements of virtue, on one hand, and the standard definitions of personality, on the other, is also an issue with this potential answer to the capaciousness of personality.

The third answer is that personality traits and virtue traits are similar, but distinct concepts. This article presents conceptual and empirical arguments for this similarity in seeing traits as a genus concept that includes the species of personality and the species of virtue. We argue for this answer because we take seriously the differences in these two types of traits and believe that the differences are sufficiently significant to require distinct trait concepts.

The fourth answer is that personality and virtue are unrelated. We dismiss this answer because there are many studies that indicate empirical relationships between personality and virtue traits and the conceptual similarity of the two forms of traits should not be ignored. Theories of personality dimensions and virtue traits also suggest their relationship as enduring traits that interact with situations and roles and are related to similar outcome variables. Ultimately, we conclude that the preferred relationship between personality and virtue, pending conceptual and empirical results indicating otherwise, is the genus-species relationship of trait as a genus and personality and virtue each being a species within that genus. We begin by surveying extant theoretical accounts of personality.

## 2. Accounts of personality

In the complex and multi-dimensional research on personality, the relatively stable and enduring aspects of the individual are thought to distinguish one individual from another. Personality dimensions are also expected to predict individuals’ future behavior by clarifying their likely behavioral tendencies, emotional responses, and cognitive processes. Extensive research has confirmed that there are some basic personality dimensions that pick out important individual differences (John and Robins, 2022) and are predictive of many aspects of life (Hurtz and Donovan, 2000; Paunonen and Ashton, 2001; Lamers et al., 2012).

### 2.1. Personality stability

The long-term stability of personality has ample evidence behind it (Roberts and Damian, 2019), and this stability was, for decades, a key assumption for most personality psychologists. One representation of this view is that the strength of the rank ordering of personality dimensions appears to increase as individuals age, which Roberts and DelVecchio (2000) termed the cumulative continuity principle. Roberts and Damian (2019) asserted “that the cumulative continuity principle is one of the most robust, replicable, and consistently supported patterns of personality development” (p. 155).

In recent decades, however, a debate has emerged in personality psychology about the degree to which personality traits are stable and consistent across different situations compared to how context-dependent and subject to change they are. Several models have attempted to explain the apparent combination of stability and variability. At one extreme, personality is studied as a consequence of circumstances in which behavior is likely to be influenced in a specific direction. For example, researchers could try to find the conditions that tend to cause people to act in a particular way, such as conforming to group norms, retaliating against an attacker, or feeling closely attached to another person (Ashton, 2022). At the other extreme, many researchers focus on stable personality characteristics, such as the Big Five (openness to experience, conscientiousness, extraversion, agreeableness, and neuroticism; Costa and McCrae, 2010) or the HEXACO (honesty-humility, emotionality, extraversion, agreeableness, conscientiousness, openness to experience; Ashton et al., 2014). These approaches generally rely on self-report scales of the relevant dimensions that have been thoroughly psychometrically assessed. In between these two extremes is a more dynamic approach to personality that focuses on a set of dynamic social-cognitive systems, internal to persons, such as desires, beliefs, values, goals, expectancies, emotional states, and so on, that produce intra-individual variability in people’s responses to situation variables. This approach was famously developed by Mischel and Shoda (1995) and Mischel (2004) and is called the cognitive-affective personality system (CAPS). In recent years, integrations of the structuralist/dimension approach and the process approach have emerged, such as whole trait theory (WTT; Fleeson and Jayawickreme, 2015) and the three-tiered framework of personality (TTFP; McAdams, 2015). These approaches incorporate both traits (from the Big Five or HEXACO) and within person social cognitive processes into whole traits or tiers of personality.

### 2.2. A brief history of the trait concept

The idea of a trait-based approach to personality can be traced back to Allport (1937). He defined traits as “a generalized and focalized neuropsychic system” (p. 313) that underlies consistent patterns of behaviors across situations (John and Robins, 2022). Allport identified 18,000 terms that can be used to describe personality and worked from there to develop what he referred to as the fundamental categories of personality traits. Several other psychologists have contributed to the early trait-based approach, including Cattell (1957), who developed the 16PF (16 personality factors) theory, which identifies 16 primary dimensions of personality, and Eysenck (1990), who developed the PEN (psychoticism, extraversion, neuroticism) model. These models and others have sought to identify the core traits that define personality and have contributed significantly to our current understanding of personality (John and Robins, 2022).

Perhaps the best-known approach to personality, the Big Five trait model proposes that personality can be described in terms of five broad dimensions: openness to experience, conscientiousness, extraversion, agreeableness, and neuroticism (McCrae and John, 1992). The NEO-PR-3 inventory, which has been lauded as the best validated measure of Big Five (John et al., 2008), includes six facets for each trait. For example, agreeableness is broken up into trust, straightforwardness, altruism, compliance, modesty, and tender-mindedness (Costa and McCrae, 2010).

The Big Five traits have strong predictive power for a wide range of outcomes in a variety of domains. For example, studies have found that individuals high in Conscientiousness tend to have better health outcomes (Lamers et al., 2012), are more likely to succeed academically (Paunonen and Ashton, 2001), and are more likely to be successful in the workplace (Hurtz and Donovan, 2000). Despite being developed several decades ago, the Big Five model remains highly relevant today. One reason for this is that the model has been assessed successfully across many cultures and languages, making it a global tool for understanding human personality (Schmitt et al., 2007).

Ashton and Lee (2005) proposed the HEXACO model as an alternative to the Big Five model after identifying a lack of nuance in agreeableness. combining the straightforwardness and modesty dimensions within agreeableness, Ashton and Lee (2005) named honesty-humility as a distinct sixth dimension. This altered both agreeableness and conscientiousness, compared to the Big Five, but left extraversion, neuroticism (emotionality), and openness to experience virtually identical in the two models. Although the Big Five model of personality has long been the standard in personality research, Ashton and Lee presented the HEXACO model as a refinement of this approach by providing additional insight into the way in which personality manifests itself in individuals through moral behavior. Specifically, the honesty-humility dimension of the HEXACO model has been linked to moral virtues such as honesty, fairness, and humility which are essential for building strong relationships and contributing to a just society. For example, Hilbig and Zettler (2009) found that individuals who score high in honesty-humility are more likely to display prosocial behaviors such as fair allocation of resources. With this brief outline of the personality domain, we turn now to the possible relationships between personality and virtue.

## 3. Personality as an all-inclusive framework for individual differences

An all-inclusive position on personality would suggest that all traits, characteristics, and individual differences should be considered in the study of personality, and that no one trait or dimension can fully capture the complexity of human personality on its own. That is, there is no core “personality” available, only more or less well integrated facets. This perspective emphasizes the importance of considering the full range of individual differences in personality, including both stable and dynamic factors, across multiple levels of analysis. Such an inclusive approach emphasizes the consistency of individual differences in behavior across situations. That is, people are comprised of certain traits that predispose them to think, emote, and behave consistently across contexts.

In the early days of personality psychology, Warren and Carmichael (1930) made the sweeping claim that “personality is the entire mental organization of a human being at any stage of his (*sic*) development. It embraces every phase of human character: intellect, temperament, skill, morality, and every attitude that has been built up in the course of one’s life” (p. 333). Few personality psychologists have been so explicitly all-inclusive in their definitions of personality. Allport (1937), for example, said that personality traits are “generalized and personalized determining tendencies—consistent and stable modes of an individual’s adjustment to his environment” (p. 328). His definition is ambiguous about its inclusiveness. This more ambiguous approach is also evident in a subsequent definition: “personality refers to those relatively stable and enduring aspects of the individual which distinguish him from other people, and at the same time, form the basis of our predictions concerning his future behavior” (Wright et al., 1970, p. 511). Finally, in describing the Big Five, McCrae and John (1992) stated rather neutrally that “the basic dimensions of personality…(are) the most important ways in which individuals differ in their enduring emotional, interpersonal, experiential, attitudinal, and motivational style” (p. 175). Although there is some variability in these definitions, they have several features in common: (1) an emphasis on individual differences, (2) an emphasis on stability over time within persons, and (3) vagueness in their inclusiveness (except for Warren and Carmichael, 1930).

The first commonality (between persons differences) is definitional. If there were no reliable between persons differences in personality characteristics, then personality would be meaningless and would be incapable of explaining anything. The second characteristic is also central, for if personality characteristics were not stable within individuals, they would also fail to reliably explain behavior, affect, or cognition. This renders the continuity principle extremely important, and its firm foundation is bedrock for personality psychology. Traditional long-term longitudinal studies have supported the stability of personality characteristics over time (Roberts and Damian, 2019).

The importance of traits was famously questioned by Mischel (2004) and more recently by Doris (2002). Both critiques relied on questioning the degree to which situational factors (e.g., ambient noise or bystanders) were more important influences on behavior than stable traits. Extensive research has suggested that minor situational factors do influence behavior (e.g., Fischer, et al., 2011). Mischel and Shoda (1995) proposed the CAPS Model to account for situational influence and Doris proposed doing away with traits altogether. Although situational factors are clearly important, we have already noted substantial evidence for the importance and stability of personal characteristics.

A strong empirical counter to these the trait skeptics comes from Fleeson (2007) and Fleeson and Gallagher (2009), who have introduced a density distribution approach to personality research. They have studied many traits in experience sampling studies, wherein they ask participants to report on their activities multiple times per day for a period of weeks. When individuals report about trait-related behavior, it provides a distribution of trait reports over time. They have reported very strong within persons consistency over time (correlations ranging from 0.7 to 0.9), for both personality characteristics (Fleeson and Gallagher, 2009) and virtue traits (Meindl et al., 2015). This demonstrates the within person consistency over time necessary for a trait. Fleeson and colleagues have also reported a great deal of within person variability despite this stability. They suggest that the within person variability is largely systematic and due to the influence of situational factors. Research has clearly corroborated this interpretation (e.g., Fleeson and Law, 2015). The experience sampling studies support the importance of traits, but they do not tell us about the relationships between personality and virtue.

Fleeson and Jayawickreme (2015) have proposed an integrative theory of personality called whole trait theory (WTT). This approach includes both traditional traits as descriptions of personal characteristics that can partly explain behavior, but also social cognitive processes that help to explain why behavior takes the form it does in specific situations. They apply this theory to both personality (Fleeson and Gallagher, 2009) and virtue traits (Jayawickreme and Fleeson, 2017). They consider virtue traits as part of personality, but they do not suggest that virtues can be fully subsumed in personality traits. This proposal has been seconded by Wright et al. (2021).

Although we are not aware of anyone who explicitly asserts that virtues are inseparable from personality, we think it worth considering this view of their relationship as a potential option. After all, this would be the most parsimonious way to understand many personal characteristics, and such an approach would locate the study of virtues within a well-established domain of scholarship.

There are four reasons that virtue theorists will reject the complete subsumption of virtues within personality. First, most virtue theorists see virtues as inherently moral characteristics, in that virtues are deemed desirable and worthwhile. This differs from personality in that scholars do not directly portray personality dimensions as inherently desirable or worthwhile (although there may be implicit valuing of some characteristics). Second, virtues are understood as acquired traits that are cultivated intentionally and by choice, whereas personality dimensions are generally considered to be based on biological tendencies. Personality scholars’ views vary from seeing personality dimensions as “purely descriptive concepts to biologically based causal concepts” (John and Srivastava, 1999, p. 130), but very few would deny that individuals are born with the rudiments of personality dimensions and that those dimensions generally unfold without intentional effort over time. Although the manner of virtue acquisition remains a matter of debate and research, virtues are viewed by most as characteristics that individuals acquire agentically because individuals see them as desirable and worthy (e.g., Fowers, 2005; Snow, 2015). Third, virtues are seen by many scholars as the characteristics that best promote a good human life, which is one of the sources of their value (e.g., MacIntyre, 1984; Fowers et al., 2021).^1^ In contrast, few, if any personality theorists recognize a necessary theoretical link between personality dimensions and a good human life. Finally, most virtue theorists recognize the centrality of practical wisdom in deciding what constitutes a virtue in a particular situation an in harmonizing the virtues toward a good human life (e.g., Darnell et al., 2019; Wright et al., 2021). For example, on an occasion wherein it may be appropriate to give a gift, one must choose whether it is proper for one to give a gift, what gift would be fitting, how to present it, and so forth. On many occasions, some people would do well to give a gift, but it would not be fitting for others to do so, and some gifts might be too miserly whereas others might be excessive, given the circumstances. In contrast, no extant personality theory includes a concept resembling practical wisdom.

The difficulty in fully subsuming virtues within personality is that these four features of virtues create very difficult choices for scholars of both personality and virtue. In order to combine these research domains, one of two things would need to happen. On the one hand, virtue theorists would have to be comfortable dispensing with these four features or, on the other, personality theorists would have to be comfortable including them in personality theory and research. Virtue theorists are generally unlikely to allow these features of virtues to be hived off because they tend to see the features as definitive of virtues. On the other hand, the inherently moral nature of virtue will be unpalatable for most personality theorists because they tend to favor a strict fact-value dichotomy. The field of personality research virtually began with Allport’s (1937) famous couplet that seemed to divide personality and virtue: “*Character is personality evaluated, and personality is character devaluated*.” (p. 52, italics in original). No personality theory includes provisions for practical wisdom, and it is very difficult to see how it can be included because it is generally seen as a meta-virtue with clear connections to a good human life (Fowers, et al., in press). Both sets of choices seem unpalatable to researchers of either personality or virtue. Nevertheless, virtue science continues to expand despite the lack of a consensual answer to the question of how personality and virtue are or are not related.

## 4. Personality as inclusive of virtue only

It is apparent that the same four features will render the inclusion of virtue alone within personality theory problematic and for the same reasons. In addition, personality theorists and researchers seldom include virtues in their publications, which suggests that most do not see virtues as relevant or important to personality. Despite this *de facto* exclusion, there are some advocates of incorporating virtues into the personality domain that we have already mentioned. Jayawickreme and Fleeson (2017) explicitly advocate for this inclusion, and they suggest that WTT provides the needed conceptual basis for the inclusion. It is instructive to explore their proposed integration. These authors argue that personality and virtues have many similarities, including their individual differences, stability, value in predicting subsequent behavior and outcomes, and amenability to very similar research methods. They also suggest other similarities, such as being volitionally changeable and being explained by social-cognitive processes. We agree with them about these similarities, but we also recognize important differences that they seem to elide, including the four features discussed in the last section. Jayawickreme and Fleeson make no provision for how those features would be included, excluded, or altered so that virtues could be incorporated into WTT.

Wright et al. (2021) also champion WTT as a suitable theoretical vehicle for personality and virtue. They see the same important similarities between the two sets of constructs as Jayawickreme and Fleeson (2017), but Wright and colleagues differ in making some provisions for two key features of virtues: moral content and practical wisdom. To be sure, many authors have recognized the moral content of personality dimensions (McCrae and John, 1992; Ashton and Lee, 2005; McAdams, 2015; Jayawickreme and Fleeson, 2017), but many more do not, and those who do see morality as part of personality seldom take it as a core feature of personality.

Because Wright et al. (2021) make efforts to accommodate virtue theory in WTT, their integration is also worth exploring. They maintain that virtues are moral traits, whereas personality traits have moral elements but are not inherently moral. They also clarify that although some elements of traits support virtue, others are core to virtue (Wright et al., 2021). For example, gregariousness may make it easier to be publicly generous, but one need not be gregarious to practice generosity. The inclusion of morality in virtues only goes so far for Wright and colleagues, because they demurred about the relationship between virtue and a good human life because this is a contentious question. We agree that there are different views on this relationship, but we see it as too central to virtue to set aside. Wright and colleagues also neglect to clarify how the inherent morality of virtues can be integrated with the tangential morality of personality dimensions.

Wright and colleagues explicitly incorporated a robust portrayal of practical wisdom as central to virtue in their discussion of WTT. They located practical wisdom as a social-cognitive mechanism. Although they acknowledge that WTT has not explicitly incorporated practical wisdom, they contend “that WTT is a hospitable empirical framework that can accommodate elements of phronesis [practical wisdom], among other mechanisms, in explaining how trait-relevant stimuli are perceived” (p. 81). Unfortunately, they did not clarify what practical wisdom’s place is among the other social-cognitive mechanisms or personality traits. This silence raises many questions. Is practical wisdom a higher order operation that guides the other mechanisms or is it just one among many? Does practical wisdom effect the integration of social-cognitive mechanisms such that their operation trends toward excellence? How do Wright and her colleagues keep practical wisdom from becoming a homunculus that guides trait expression? Is practical wisdom a trait itself with its own descriptive side or just a mechanism that helps explain the other trait manifestations? Does practical wisdom also regulate personality trait expression or only virtue trait expression? These authors may have answers to these questions, but they have not provided them explicitly. The bottom line is that Wright and colleagues have offered a partial integration of personality and virtue, but this does not suffice from our perspective, and we remain unconvinced that WTT is fully consistent with virtue.

McAdams (2009, 2015) has consistently discussed morality as a central element of personality in his TTFP, making his attempt at integrating personality/virtue interesting. He frequently discusses meaning, purpose, and belonging as central goods for humans, at least in Western culture. In addition, for McAdams, agency and morality do not differentiate personality and virtue, and he recognizes that the attempt to create pure descriptions of personality traits have built moral concerns into those traits. McAdams explicitly frames personality as an integrative narrative and states that narratives are inextricably moral because they involve choices about pursuing what is valued. This is rather close to the virtue ethics idea that one’s characteristics can be shaped into a valued and worthwhile way to live. Although the TTFP is easily understood in moral terms, its version of living well is not directly tied to virtues as the characteristics that make a good life possible. McAdams (2009) does discuss personality traits and characteristic adaptations as having clear roles in fostering a good life, but these connections are rather cursory and not explicitly and sufficiently made from a virtue perspective. The other gap, from a virtue theoretical perspective, is that TTFP has no provision for the meta-virtue of practical wisdom.

One possibility is that future theory and research may indicate that personality and virtue traits are sufficiently similar that integrating them is advisable. We do not believe that we have reached that time yet, however. Despite Wright et al. (2021) and McAdams (2015) impressive efforts, we do not believe that any personality theory has been sufficiently enriched or elaborated to accommodate virtue theory. A less demanding version of virtue theory may be more easily accommodated, but most virtue theorists make stipulations that contemporary personality theory and research are unlikely to accommodate. We recapitulate the four main sticking points here.

First and foremost, the moral character of virtues is central. With a few exceptions (e.g., McAdams, 2009; Jayawickreme and Fleeson, 2017), academic psychologists are rather uncomfortable with inherently moral concepts, believing that they can provide an ostensibly objective description of facts about human behavior. There are significant doubts about whether such a value neutral approach is possible (Fowers, et al., in press; Brinkmann, 2011). We believe that evaluative commitments are rampant and unavoidable in psychology because we see humans as inextricably moral creatures and human activities (including social science) as generally imbued with moral purpose and import (Fowers, et al., 2022). The debate about the place of morality in psychology is as old as the discipline and remains unresolved. Until morality can be relatively easily incorporated into psychological theories, we doubt that virtues can be meaningfully and fully integrated with personality or any other traditional psychological research topic. As noted, Wright et al. (2021) have made a good beginning in proposing the sort of interdisciplinary integration that might work. Although promising, their framework has not been accepted by many psychologists or philosophers.

Second, virtue ethics emphasizes humans’ agency, in that virtues must be chosen and intentionally cultivated (Fowers, et al., 2021; Wright et al., 2021). Virtues must be cultivated through repeated practice, and this leads to habitual modes of action. Therefore, agency is central to virtue. With a few exceptions (e.g., Martin and Gillespie, 2010; McAdams, 2015), psychologists are far more comfortable with discussing causal relations than telic or agentic relations. This discomfort with telic or agentic relations is due to historical choices favoring causal accounts because they seemed more “scientific” by mimicking the physical sciences. The debate about whether humans are best conceived as “driven” by a nexus of causal forces has been active throughout the history of psychology, as seen in numerous cogent critiques (e.g., Richardson et al., 1999; Martin and Gillespie, 2010). This debate notwithstanding, the frequent use of causal language and the deterministic focus of much of psychology predominate. This is clear in the view that personality dimensions tend to emerge without intentional effort, making personality more focused on causal rather than agentic sources. We will not attempt to resolve the agency/determinism debate here, but we note the frequent use of causal expressions even as the WTT is presented as a contender for an integrative view of personality and virtue. Jayawickreme and Fleeson (2017) stated, for example, that WTT “postulates a number of processes causally implicated in the manifestation of behavior” and “that several such processes are the determinants of behavior” (p. 122). This causal language is far from accidental. Although these authors occasionally discuss individual choice, they do not elaborate on how agentic relations are central to virtue or on how causal and agentic sources of behavior can be integrated. In our view, in insisting on causal explanations, psychologists do not have the tools to do justice to agentic relations. This renders it impossible to integrate virtue with personality or any other traditional psychological research topic.

Third, we see virtues as inherently teleological in that cultivating and practicing virtues is about crafting a good human life. We emphasize that both virtuous activity and a good human life are modes of living that are seen by many as mutually conducive (MacIntyre, 1984; Fowers, et al., 2021). Many domains of psychological theory and research contain telic thinking, such as goal pursuit (e.g., Baumert et al., 2017) and developmental psychology (e.g., Sokol et al., 2015), but even these domains are seldom framed in terms of ultimate aims such as a good life. This is likely a form of value neutrality due to the potential controversies about what comprises a good life. To avoid these controversies, some virtue theorists have preferred to remain silent on the relationships between virtues and a good human life. We believe that these relations must be empirically verified, but we also see them as too important to ignore. We hasten to add that there are many ways to live well as a human being, so adopting a good life as a goal does not amount to imposing one’s values.

Finally, practical wisdom is a centerpiece for most neo-Aristotelian virtue theories (e.g., Fowers, 2005; Darnell et al., 2019; Fowers, et al., in press), although there are debates about what constitutes it. Practical wisdom is seen by many as a meta-virtue that guides the ways that virtues show up and is vital to the relations between virtues and a good human life (e.g., MacIntyre, 1984; Fowers, et al., 2021; Kristjánsson et al., 2021). Others see practical wisdom as necessary for virtue but view it as a set of functions rather than seeing it as a trait (e.g., Wright et al., 2021). Neither the authors of WTT nor of TTPF have themselves incorporated a robust version of practical wisdom. As noted, Wright et al. (2021) incorporated practical wisdom in their reinterpretation of WTT, and we see their work as a helpful reformulation. Nevertheless, their version of WTT left significant questions unanswered, so their reinterpretation falls short in our view. Practical wisdom is both moral and teleological. It is moral because practical wisdom is always related to morally better and worse courses of action and aims. Practical wisdom is telic because it is ultimately aimed at the end of a good human life. Until practical wisdom can be fully integrated in psychological theory, we doubt that many virtue theorists will be satisfied with integrations of virtue with personality or any other psychological construct.

The similarities between personality dimensions and virtues are multiple, such as conceptualizing and measuring them as traits with individual differences, intraindividual stability and variation, that they interact with situations, change over time, and are partly comprised by life narratives and cultural influences. Yet the key differences between standard psychological theory and research and conceptions of virtue (e.g., the inherence of morality, the key role of agency, focus on a good life, and the integral place of practical wisdom) render a ready integration impossible at this time.

## 5. An account that sufficiently differentiates virtue and personality

We have, to this point, considered two possible ways that virtues and personality traits can be combined. The first and simplest approach folded all stable individual differences into personality, which could allow virtues to be subsumed within the personality construct. Although this view seems charmingly unwrinkled, it ran into basic problems, because the personality domain does not appear to be sufficiently capacious to encompass core features of virtues.

The second approach we considered was limiting the integration to personality and virtues. This approach improves on the first by restricting personality as a construct to a more plausible remit, while retaining the convenient subsumption of virtue traits (*qua* traits). However, this approach also introduces similar difficulties, because contemporary personality theory and research are insufficiently capacious.

### 5.1. Conceptual arguments for personality-virtue differentiation

We now consider a third approach, which differs from the first two by fully acknowledging the differences between the personality and virtue constructs, which augurs against the complete subsumption of virtue traits. Instead, we agree that, although personality and virtue share certain features, they have substantial differences which render it difficult to collapse them into a single conceptual frame. Accordingly, we use a biological metaphor that suggests considering them each as species of a *trait* genus. That is, their commonalities spring precisely from the fact that personality and virtue both instantiate *traits* (i.e., relatively stable dispositions within individuals with varying degrees of situational activation). But their differences are sufficient to justify a conceptual distinction at the level of species.

Before defending this position, we will make clear what it entails. First, a species-genus relationship requires a *family resemblance* between personality and virtue traits, where what is shared between them hinges on their location in the genus of trait. Second, a species differentiation requires that each collection of traits possesses characteristic differences preventing one from being entirely subsumed in the other.

To begin by accounting for essential differences, consider that virtue traits, on most practical accounts, require intentional cultivation to be considered full instantiations of virtue (cf. Snow, 2015; Upton, 2017). Contrast this cultivation requirement with personality traits, which do not turn on cultivation, and on some traditional accounts are precisely those traits of an individual which are less responsive to cultivation (Costa and McRae, 1986). A critic of this differentia could point out that virtue traits, being conceived as desirable, have generated a science of cultivation, but that personality traits could also be viewed as desirable, and also generate cultivation efforts. This critique is supported by research indicating that an intention to increase a given personality dimension has been observed to precede an increase in daily behaviors associated with that personality dimension (Hudson and Fraley, 2015). Although this parallel seems somewhat stretched, it cannot be dismissed. Insofar as both virtues and personality dimensions are dispositions, this indicates a family resemblance among them. Insofar as virtues are intentionally cultivated, whereas personality dimensions emerge on their own, this developmental variation differentiates them.

As we have noted, this differentia can be further refined by observing that virtue traits are generally defined with respect to specific goods, or worthwhile ends (Aristotle, 1999; Carr et al., 2016). This teleological quality distinguishes virtue traits from most accounts of personality traits, at least at the conceptual level, because personality traits are seldom assumed to have a teleological structure. This is not to say that certain personality traits cannot, in fact, assist individuals to accomplish goals, but that the class of personality traits does not share a family-resemblance with virtues in the form of orientation toward a consistent set of goals. A critique of this differentia might proceed along the suggestion that traditional personality traits (such as conscientiousness) do in fact have characteristic goods as their end, and that they are simply not discussed as such. More incisively, it might be that virtue traits are simply personality traits conceived in terms of their ends, where the difference between the two groups is one of emphasis, rather than kind. This is also evident empirically in the strong relationships that have been found between personality and goals (McCabe and Fleeson, 2012; Baumert et al., 2017). Again, this stretches the concepts of personality and virtue, but this position has some plausibility.

In response to this critique, we can develop our differentia even further by considering that virtue traits require harmonization for their proper exercise. That is, due to their teleological structure, the additional capacity of practical wisdom is necessary to adjudicate between competing virtue desiderata (Kristjánsson et al., 2021). Although personality traits may present competing demands on the person, resolving these competing demands does not require an additional capacity, in part because personality traits do not, as a class, have essential relationships to each other. That is, personality traits are generally conceived as modular and separable rather than in terms of the harmonization that seems necessary for aims related to human goods. The unifying node of practical wisdom, and the implicit suggestion that virtues can be harmonized in the service of a good human life, strongly distinguishes the cluster of virtue traits from personality traits and is a defensible differentia in service of the species distinction between the two.

It is worth considering that some theorists believe there is an inextricable relationship between virtue and a good human life (MacIntyre, 1984; Fowers, 2005). The argument is that the concept of virtue is central to understanding and pursuing human flourishing. From this perspective, virtues are habits or dispositions that enable individuals to pursue a good life, meaning that there is an inextricable relationship between the two. This relationship has been contested by some (e.g., Driver, 2001; Tessman, 2005). Nevertheless, many virtue theorists argue that being virtuous leads to a kind of flourishing which is not otherwise accessible (e.g., Fowers, et al., in press; Kristjánsson et al., 2021). Once again, it is possible to contend that some personality traits might also conduce to flourishing, but, to our knowledge, such an argument has not been made. If it were, then virtue traits might share a family resemblance with at least those personality traits which happen to conduce to flourishing.

Finally, there is also a version of virtue ethics that identifies virtues with skills (Annas, 2011; Stichter, 2018), such as woodworking, jazz, or chess. This sophisticated interpretation of virtues draws on the domain of expertise to flesh out the metaphor of virtues as skills that Aristotle (1999) also used. The skills perspective is especially helpful with the cultivation of virtues, which is thought to follow the pattern of skill cultivation, through repeated practice. The skill conception differentiates virtues and personality dimensions because personality dimensions have not been conceptualized as skills. In addition, greater skillfulness and expertise are generally valued because they indicate an achievement, which is a poor fit for most personality models. We encourage readers to explore this version of virtue ethics, but we will not address it further in this paper.

Now that we have presented a satisfying differentia (that of *phronetic* coordination aimed at a larger good), we must examine similarities shared between personality and virtue traits to justify their placement in the genus of the trait family. That personality traits and virtue traits are both species of the trait genus suggests that they would often correlate with one another, and research has documented such relationships, as we discuss presently.

### 5.2. Empirical evidence for personality-virtue differentiation

An early entry in virtue measurement was made by Seligman et al. (2004), with their creation of the value in action (VIA) framework and the value-in-action inventory of strengths (VIA-IS). The VIA-IS remains one of the most widely used self-report instruments of virtues in adults (McGrath, 2019). The VIA-IS assesses 24-character strengths across six overarching virtues of courage, justice, humanity, temperance, transcendence, and wisdom. Character strengths are defined as universal, trait-like, and morally valued dispositions that lead to optimal psychological outcomes. In contrast to the hypothesized six-factor structure of the VIA-IS, “no single factor structure encompassing the entire set of strengths has emerged across measures of the VIA model” (McGrath et al., 2020, p. 118). Several studies of the relationships among personality dimensions and the original or factor analysis derived VIA-IS components have found moderate correlations between them (Noftle et al., 2011; Furnham and Ahmetoglu, 2014; McGrath et al., 2020). In the McGrath and colleagues’ study, correlations (absolute value) of the VIA-IS scales ranged from 0.30 to 0.62 with the NEO-PI-R (Big Five) and from to 0.47 to 0.78 for the HEXACO.

The VIA-IS has been widely used to measure positive character attributes in different international populations (Biswas-Diener, 2006; Park et al., 2006; Linley et al., 2007; Peterson et al., 2007). Although there is similarity in responses across cultures, the VIA-IS was derived from a questionable universalism (McGrath, 2015; Fowers et al., 2023). The VIA-IS has also been subject to criticism due to its psychometric inconsistencies. Even within the American context in which it was developed, the posited six-factor structure of the VIA-IS has been found to vary drastically (Khumalo et al., 2008; Martínez-Martí and Ruch, 2017; Diez et al., 2023).

Studies with other virtue measures have produced similar results. For example, research on the Justice Sensitivity scales revealed correlations with the Big Five ranging from 0.00 to 0.36 (Schmitt et al., 2005; Rothmund et al., 2014). Schmitt et al. (2010) found correlations among the 30 personality facet scales of the NEO-PI-R (each Big Five dimension has six facet scales) and the four forms of Justice Sensitivity (victim, observer, beneficiary, and perpetrator sensitivity) to be below 0.30 with German participants. Breen et al. (2010) also reported only moderate correlations (ranging from 0.17 to 0.59) between measures of gratitude and forgiveness with the Big Five dimensions. McCullough et al. (2002) also reported two measures of gratitude had low to moderate correlations with the Big Five dimensions, ranging from 0.23 to 0.52. Wood et al. (2009) found similar results, with correlations between a gratitude measure and the 30 facets of the Big 5 (NEO-PI-R) ranging from 0.01 to 0.51. Finally, Brown (2003) found mild to moderate correlations between trait forgiveness and Agreeableness (*r* = 0.43) and neuroticism (*r* = −0.39), but no relationships with the other Big Five dimensions. These results are high enough to evidence overlap among personality dimensions and virtues and low enough to suggest distinctiveness across the two categories of measurement.

Another way to assess the distinctiveness of personality and virtue measures is whether virtue measures have incremental validity vis a vis personality assessment. In other words, do virtue measures predict relevant criterion measures after statistically controlling personality dimensions? This form of evidence has been used frequently to support the claim that virtue scales are different from, and in some cases, superior to, personality measures in predicting important outcomes.

For example, in predicting job performance, character strengths and general mental ability were found to have incremental validity after controlling Big Five dimensions (Harzer et al., 2021). In a study of students and teachers, some character strengths (e.g., love of learning and perseverance) were consistently related to achievement and positive learning experiences (flow and enjoyment) after controlling both cognitive ability and personality dimensions (Wagner et al., 2020). McGrath et al. (2020) reported that, in over 90% of their analyses, the VIA-IS scales evidenced incremental validity for several criterion measures vis a vis the Big Five and HEXACO personality measures. The justice sensitivity scales have also been found to predict outcomes above and beyond personality facets (Rothmund et al., 2014). McCullough et al. (2002) reported that gratitude also significantly predicted positive affect, well-being, and prosocial behaviors and traits after controlling the Big Five dimensions. Wood et al. (2009) found that gratitude had incremental validity over the 30 facets of the Big Five in uniquely predicting satisfaction with life. Other studies have also controlled the Big Five and found that gratitude indicated incremental validity with criterion measures (Wood et al., 2009; Morgan et al., 2017). Finally, two experimental studies found that self-report virtue traits of kindness and fairness predicted kindness and fairness behavior (respectively) after controlling the Big Five dimensions of agreeableness and conscientiousness (Lefevor and Fowers, 2016; Fowers et al., 2022).

The species-genus relationship is a conceptual move that can resolve the tension generated between virtue traits and personality traits as psychological constructs. We believe that the species-genus relationship is an efficient way to respect both the similarities and differences of personality and virtue traits within a single conceptual scheme. Based on the available evidence, this species-genus relationship is fitting for both the Big Five and the HEXACO with respect to many available measures of virtues. We have argued that attempts to subsume virtue traits into the personality trait construct typically fail, which indicates that the differences between the constructs may be too substantial for full integration. Our conclusion, therefore, is that those differences must be taken seriously.

## 6. An account with personality and virtue as entirely distinct

A final possible way to understand personality and virtue is that they are entirely distinct. Although there is reasonable disagreement about subsuming virtues into the study of personality traits, few would go as far as to deny any relationship between personality and virtue. In fact, there are moral elements incorporated in how several personality traits are defined. Two of the Big Five personality dimensions (Conscientiousness and Agreeableness) draw upon moral-laden terms to describe the facets of each trait (NEO-PR-3; Costa and McCrae, 2008). Conscientiousness is based on the six facets of competence, order, dutifulness, achievement striving, self-discipline, and deliberation. similarly, agreeableness consists of trust, straightforwardness, altruism, compliance, modesty, and tender-mindedness. These facet terms include moral elements, thereby calling into question the validity of drawing a clear distinction between virtue and personality. The intermixing of personality and virtue is further confounded by the addition of the sixth honesty/humility dimension in the HEXACO model, “defined by terms such as sincere, fair, and unassuming versus sly, greedy, and pretentious” (Ashton and Lee, 2005, p. 1324). The moral overtones of this definition were intentional, as seen in Ashton and Lee’s use of terms such as modesty, fairness, and honesty.

In addition to the integration of moral or virtue elements into personality measurement, abundant research indicates multiple correlational relationships between Big Five and HEXACO measures of personality, on the one hand, and morally imbued characteristics, on the other (Fleeson et al., 2014). For example, research indicates that young adult moral exemplars score high on Agreeableness (Matsuba and Walker, 2004), Agreeableness predicts physician empathy (Song and Shi, 2017), Extraversion is positively related to group cooperation (Ross et al., 2003), and that conscientiousness, extraversion, agreeableness, and emotional stability relate to helping behavior at work (King et al., 2005). Personality dimensions have also been found to correlate with morally negative characteristics. For example, research indicates narcissism correlates positively with Extraversion and negatively with the honesty-humility factor of the HEXACO model (Lee and Ashton, 2005), and that low conscientiousness and agreeableness predict academic cheating (Giluk and Postlethwaite, 2015) and infidelity (Schmitt, 2004). Therefore, there is little question of whether there is a relationship between personality and moral constructs.

Finally, we reviewed many studies of the positive relationships among personality dimensions and more directly assessed virtue traits in the previous section. Any claims about the independence of personality and virtue traits run counter to a good deal of empirical evidence that suggests that they are at least moderately related. Therefore, we conclude that the two cannot be rendered distinct.

## 7. Conclusion

We have explored four possible relationships between personality and virtue traits. The question about how best to view this relationship has become increasingly pressing as virtue science has developed (Fowers et al., 2021). We discounted the first possibility, that all individual differences are redundant to personality dimensions primarily on conceptual grounds because it would be quite difficult for personality theory and research to accommodate concepts that are central to virtues (morality, agency, a good life, and practical wisdom). We could add that the empirical evidence is inconsistent with seeing virtues as entirely redundant to personality inasmuch as the correlations between measures of the two forms of traits are only mild to moderate, and virtues have demonstrated incremental validity over personality dimensions. We discounted the second possible relationship—that virtues alone can be subsumed in personality—for the same reasons. The fourth possible relationship, that personality and virtue are not related was dismissed as inconsistent with the results of every study that has examined them in tandem.

Our elimination of these three possibilities leaves only the third possible relationship, that personality and virtues share some features, but differ on others sufficiently to maintain a clear distinction between the two. We suggest that theorists and researchers ought to take *both* the similarities *and* the differences between these constructs seriously. We proffered a biological metaphor for doing this by incorporating the similarities between personality and virtue within a genus concept of traits. We view all traits as being constructs that evidence (a) between persons differences, (b) within person stability, (c) responsiveness to situational variation, and (d) relationships with important criterion measures (e.g., well-being, achievement). Within this genus, we recommend seeing personality as one species, wherein people have traits that are present in rudimentary form at birth, develop through the lifespan with or without intentional effort, and can be viewed in modular form. In a modular format, personality dimensions can be configured in any number of ways, with few entailments among them. Similarly, the species level trait of virtue would have the differentia of a trait that is intentionally cultivated and guided by practical wisdom toward a good human life, as that is conceived by agents and their communities. We see the genus/trait view as a positive way to take the similarities between personality and virtue seriously, while also avoiding glossing over the differences between the two constructs.

An additional benefit of having a two-tiered approach to traits is that there may be other species of traits that have similarities to personality dimensions but also important differences that need to be acknowledged. We do not explore these differences in this article, but two stable individual differences that come to mind are cognitive characteristics (e.g., intelligence) and psychopathological (e.g., psychopathy) traits. These constructs have important differences from the Big Five and HEXACO approaches to personality that, in our view, require acknowledgement as much as the similarities in trait constructs do.

Our aim has been to take the conceptual and empirical considerations into account and to propose a two-tiered approach to organizing an approach to personality and virtue. In doing so, we propose a genus/species understanding of personality and virtue that recognizes both similarities and differences across the two forms of traits. This will assist the budding research domain of virtue science without unduly burdening the more established personality research domain.

## Author contributions

BF conceptualized the manuscript, wrote the introduction and conclusion, and edited the entire final manuscript. LN and NK wrote a section of the manuscript and edited other sections. MS wrote a section of the manuscript. All authors contributed to the article and approved the submitted version.

## Conflict of interest

The authors declare that the research was conducted in the absence of any commercial or financial relationships that could be construed as a potential conflict of interest.

## Publisher’s note

All claims expressed in this article are solely those of the authors and do not necessarily represent those of their affiliated organizations, or those of the publisher, the editors and the reviewers. Any product that may be evaluated in this article, or claim that may be made by its manufacturer, is not guaranteed or endorsed by the publisher.

## Footnotes

^1^We hedge about virtue theorists because there are many different views among them. We are presenting what seems to us to be the majority views, but, for example Driver (2001) and Tessman (2005) would be likely to question the link between virtue and a good human life.
